# A nonparametric Bayesian approach for clustering bisulfate-based DNA methylation profiles

**DOI:** 10.1186/1471-2164-13-S6-S20

**Published:** 2012-10-26

**Authors:** Lin Zhang, Jia Meng, Hui Liu, Yufei Huang

**Affiliations:** 1 School of Information and Electrical Engineering, China University of Mining and Technology, Xuzhou, 221116, China; 2 Department of Electrical and Computer Engineering, University of Texas at San Antonio, San Antonio, TX 78249, USA; 3 Department of Biostatistics, University of Texas Health Science Center at San Antonio, San Antonio, TX 78229, USA

## Abstract

**Background:**

DNA methylation occurs in the context of a CpG dinucleotide. It is an important epigenetic modification, which can be inherited through cell division. The two major types of methylation include hypomethylation and hypermethylation. Unique methylation patterns have been shown to exist in diseases including various types of cancer. DNA methylation analysis promises to become a powerful tool in cancer diagnosis, treatment and prognostication. Large-scale methylation arrays are now available for studying methylation genome-wide. The Illumina methylation platform simultaneously measures cytosine methylation at more than 1500 CpG sites associated with over 800 cancer-related genes. Cluster analysis is often used to identify DNA methylation subgroups for prognosis and diagnosis. However, due to the unique non-Gaussian characteristics, traditional clustering methods may not be appropriate for DNA and methylation data, and the determination of optimal cluster number is still problematic.

**Method:**

A Dirichlet process beta mixture model (DPBMM) is proposed that models the DNA methylation expressions as an infinite number of beta mixture distribution. The model allows automatic learning of the relevant parameters such as the cluster mixing proportion, the parameters of beta distribution for each cluster, and especially the number of potential clusters. Since the model is high dimensional and analytically intractable, we proposed a Gibbs sampling "no-gaps" solution for computing the posterior distributions, hence the estimates of the parameters.

**Result:**

The proposed algorithm was tested on simulated data as well as methylation data from 55 Glioblastoma multiform (GBM) brain tissue samples. To reduce the computational burden due to the high data dimensionality, a dimension reduction method is adopted. The two GBM clusters yielded by DPBMM are based on data of different number of loci (P-value < 0.1), while hierarchical clustering cannot yield statistically significant clusters.

## Background

DNA methylation profiles has become an alternative molecular footprint for classification. It occurs in the context of a CpG dinucleotide. It is an important epigenetic modification, which can be inherited through cell division. In this chemical modification of the cytosine nucleotide, the 5-carbon position is enzymatically modified by the addition of a methyl group such that cytosines can occur in a methylated or unmethylated state. CpG islands are usually not methylated in normal tissues but frequently become hypermethylated in cancer [1]. This hypermethylation is associated with gene silencing [2] and plays an important role in the inactivation of tumor suppressor genes. Most CpGs or CpG regions have been found to have a bimodal distribution of methylation profiles, either hypomethylated or hypermethylated [3]. Unique methylation patterns have been shown to exist in diseases including various types of cancer [4]. DNA methylation analysis promises to become a powerful tool in cancer diagnosis, with possible applications to the choice of treatment and prognostication. The high throughput methylation profiling technology has been developed to survey methylation status of more than 1500 CpG sites for a large collection of cancer genes and been specifically targeting. Studying how the methylation profiles can be used to distinguish different subtypes of the tumor has been a focus in current cancer research. But most existing algorithms working on methylation data are from sequence level. The exact levels of methylation expression are not fully considered yet.

To this end, clustering analysis is often used to identify methylation subgroups that are distinct from one another in data [5,6]. However, the DNA methylation data presents unique challenges. First, it is not appropriate to cluster DNA methylation expressions using traditional clustering methods. The traditional k-means clustering algorithms are based on Gaussian Mixture Model (GMM) assumptions. In GMM, the individual data points are assumed to follow multivariate Gaussian distribution and thus the distance between two points can be evaluated by Euclidean distance conveniently. However, since "beta" values from DNA methylation array represent the percentage of the methylated alleles and are between 0[1], traditional GMM is no longer appropriate. Instead, a mixture of the beta distribution [7,8] would be a more accurate model. Second, a model selection process is often needed in clustering to determine the number of clusters, making the clustering analysis more complicated. A predefined number of clusters (or model) is required in the mixture distribution based methods (such as k-means). Since different number of clusters will yield different clustering results, a model selection process is desirable to determine the best number of clusters. The model selection is very different problem, whose optimal solution is of exponential complexity. The popular suboptimal solutions have been proposed that include minimum description length (MDL) and Bayesian information criterion (BIC). Although computationally efficient, these methods would fail when clusters are not well separated. The recent proposed nonparametric Bayesian methods including Dirichlet process (DP) provide an avenue that can lead to a better solution.

In a response to the aforementioned limitations, we proposed here a nonparametric Dirichlet process beta mixture model (DPBMM) method for clustering DNA methylation expression profiles produced by Illumina Infinium Beadchip. DPBMM makes use of Dirichlet process mixture to place a prior [9] on cluster assignment, thus enables automatic determination of the optimal number of clusters. To perform the analytical intractable learning, an algorithm based on Gibbs sampling and "no-gap" sampling is developed to effectively infer all the relevant variables. The proposed DPBMM method builds an infinite beta mixture model to describe DNA methylation data, which is different from the finite beta mixture model in [8]. We present a simulation study comparing its properties to RPMM (Recursively partitioned mixture model) employing BIC (Bayesian information criterior) in [8]. The results demonstrated the better performance of our proposed method. Finally, we applied the DPBMM to the methylation array obtained from 55 Glioblastoma Multiform (GBM) brain tissue samples.

## Methods

### Problem formulation

#### Model DNA methylation profiles with beta mixture distribution

**Figure 1:**
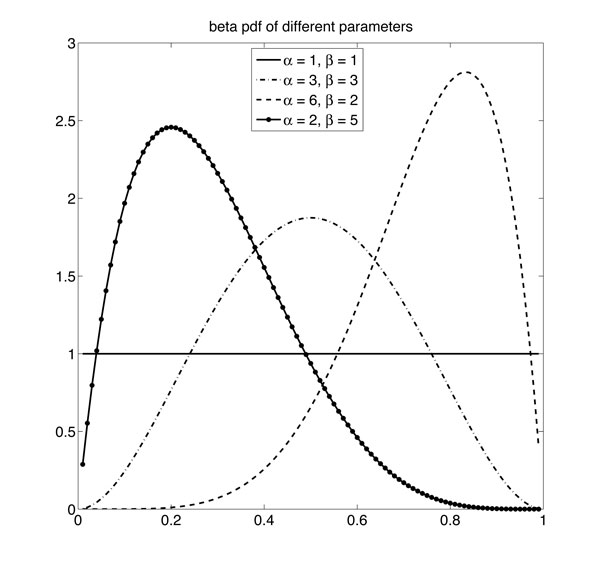
**Examples of beta distributions**. Beta densities with large hyperparameters (*α *> 1, *β *> 1) are unimodal.

For a two-color hybridization based array such as Illumina Infinium array, the measurements are associated with the percentage of the methylated alleles, which is called the "beta" values because it can be described by a mixture of beta distributions [7,10]. Since the distribution of "beta" values shows bimodalities [11], the beta distribution component in the mixture model should be convex, which means the beta distribution component should be equipped with large parameters, shown in Figure 1.

Consider the problem of clustering *n *independent DNA methylation samples, let *X *= {*X*_1_, *X*_2_, ..., *X_n_*} be the DNA methylation expressions for *n *samples. For each sample *i*, *X_i _*= {*x*_*i*1_, *x*_*i*2_, ..., *x_iL_*} be a vector of *L *continuous outcomes falling between zero and one. Suppose there exists a total *K *clusters and sample *i *belongs to cluster class *c_i _*∈ {1, ..., *K*}. Conditional on class membership say *k*, each outcome *x_il _*could be viewed as an independent identically distributed variable from a beta distribution with *α_kl _*and *β_kl_*

(1) $$f\left(x_{il}|\alpha_{kl},\beta_{kl},c_{i}=k\right)=\frac{x_{il}^{\alpha_{kl}-1}{\left(1-x_{il}\right)}^{\beta_{kl}-1}}{B\left(\alpha_{kl},\beta_{kl}\right)}$$

where $B\left(\alpha ,\beta \right)=\int_{0}^{1}x^{\alpha -1}{\left(1-x\right)}^{\beta -1}dx$ stands for the Beta function. Then, DNA methylation sample *X_i _*can be modeled by (2).

(2) $$p\left(X_{i}|\theta \right)=\overset{K}{\underset{k=1}{\sum }}\pi_{k}\overset{L}{\underset{l=1}{\prod }}\frac{x_{il}^{\alpha_{kl}-1}{\left(1-x_{il}\right)}^{\beta_{kl}-1}}{B\left(\alpha_{kl},\beta_{kl}\right)}$$

where $\theta_{l}=\left\{\alpha_{kl},\beta_{kl},\forall l\right\}$. With the limitation of large parameters for beta distribution component, *α_kl _>*1 and *β_kl _>*1. Note that due to clustering in samples, *θ_l _*and *θ_i _*for *i *≠ *l *may be equal, $\pi_{k}^{\prime }\text{s}$ represent the cluster proportion and $\sum_{k=1}^{K}\pi_{k}=1$. Now, in reality, the total cluster number *K *is not known *a priori*. We discuss next a model based on Dirichlet process to address this difficulty.

#### Dirichlet process mixture model

**Figure 2:**
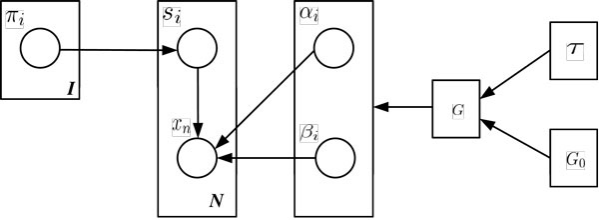
**Graphical model**. The model for the Bayesian estimation is built following the principles of graphical model.

The Dirichlet process is an nonparametric extension of the original Dirichlet distribution. Let *x_i _*be a random sample from a distribution *f *with parameters *θ_i_*. In a Bayesian formulation, the model for parameter *θ_i _*can be defined as

(3) $$\begin{array}{llll} x_{i}|\theta_{i} & \sim f\left(\theta_{i}\right)\; & & \;\\ \theta_{i}|G & \sim G\; & & \;\\ & \; & \end{array}$$

where *G *is the prior distribution of *θ_i_*. It is not always realistic to assume that *G *is of a known form and the nonparametric Bayesian models including the Dirichlet process (DP) is proposed to address this problem. Now, instead of defining a parametric form for *G*, *G *is assumed to be a draw from a Dirichlet process with a base distribution *G*_0 _and a precision parameter *τ *[12]. The model for the Bayesian estimation is also built in Figure 2 following the principles of graphical models. It can also be written as (4) with a DP prior.

(4) $$\begin{array}{llll} X_{i}|\theta_{i} & \sim f\left(\theta_{i}\right)\; & & \;\\ \theta_{i}|G & \sim G\; & & \;\\ G|\tau ,G_{0} & \sim DP\left(\tau ,G_{0}\right)\; & & \;\\ & \; & \end{array}$$

where *G*_0 _is such that *E*[*G*] = *G*_0 _and has a parametric form, *τ *measures the strength of belief in *G*_0_. The DP of mixtures (DPM) are proposed to model the clustering effect in data. Compared with other clustering models, DPM is very attractive because it allows the cluster number *K *to be *a priori *∞ and learned from the data. To capture the clustering natural of DNA methylation samples, a beta mixture model with infinite classes can be built with DPM. Let *θ_i _*= {*α_i_*, *β_i_*} be the set of parameters for each sample and note that some of them may be equal. In DPM models, each *θ_i _*is marginally sampled from *G*_0_, and with positive probability some of the *θ_i _*are identical due to the discreteness of the random measure *G*. Therefore the new value of *θ_i _*can either be one of the $\theta_{l}^{\prime }\text{s(}l\neq i\text{)}$, or *θ_i _*could be a new draw from *G*_0_. Let *K *in (2) be ∞, we assume a DPBMM for DNA methylation array.

### Inference

Let Φ = {Φ_1_, Φ_2_, ..., Φ*_K_*} denote the set of distinct $\theta_{i}^{\prime }\text{s}$, where *K *is the number of distinct elements of *θ*_1_, ..., *θ_m_*. Let *s *= {*s*_1_, ..., *s_m_*} denote cluster assignment vector, that means, *s_i _*= *l *if and only if *θ_i _*= *ϕ_l_*. Then *θ *= {*θ_i _*: *i *= 1, ..., *m*} can be reparameterized as {*ϕ*_1_, ..., *ϕ_k_*, *s*_1_, ..., *s_m_*}. Let *n_i_*, *i *= 1, ..., *K *be the number of elements *s_l _*equal to *i*. Let subscript "*-i*" stands for all the variables except the *i*-th one. The goal from a Bayesian perspective is to calculate the posterior distribution of the known parameters {Θ, *π*, *τ*}. However, the analytical expression is intractable and we instead develop a Gibbs sampling solution to obtain random samples from the posterior distribution. The key for Gibbs sampling is to derive the conditional posterior distributions of the unknown parameters. Due to the constrains on *α *and *β*, we first re-parameterize *α *as *L_α _*by *α *= *exp*(|*L_α_*|) and *β *as *L_β _*by *β *= *exp*(|*L_β_*|). Thus, we only need to sample in the range of (-∞, ∞) for *L_α _*and *L_β_*. Then the transformed *α >*1 and *β >*1. Thus, we can specify *G*_0 _as $G_{0}\left(\alpha ,\beta \right)=N\left(0,\sigma_{\alpha }^{2}\right)N\left(0,\sigma_{\beta }^{2}\right)$, where $N\left(\mu ,\sigma^{2}\right)$ represents the Gaussian distribution with mean *μ *and variance *σ*^2 ^[13]. The prior distribution of the cluster proportion *π *is the Dirichlet distribution

(5) $$\pi \sim Dir\left(n_{1}+\tau /K,...,n_{K}+\tau /K\right).$$

There are some useful expression of a Dirichlet process, such as Chinese Restaurant Process(CRP) [14,15], Stick-breaking construction [16], Polya Urn formulation [17,18], etc... Blackwell showed that Dirichlet process are discrete as they consist of countably infinite point probability masses [19]. Escobar and West [20] first showed that Markov Chain Monte Carlo (MCMC) techniques, specifically Gibbs sampling, could be used for posterior density estimation if the Blackwell-MacQueen Polya Urn formulation of Dirichlet process is used. Based on the generalized Polya urn scheme, the conditional prior distributions *s_i_*|*s*_1_, ..., *s*_*i*-1_, *i *= 1, ..., *n *and *θ_i_*|*θ_-i _*have the following forms as (6) and (7).

(6) $$\begin{array}{l} P\left(s_{1}=1\right)=1\\ \left\{\begin{array}{c} P\left(s_{i}=l|s_{1},...,s_{i-1}\right)=\frac{n_{-i,l}}{\left(\tau +i-1\right)},l=1,...,k_{i}\\ P\left(s_{i}=k_{i}+1|s_{1},...,s_{i-1}\right)=\frac{\tau }{\left(\tau +i-1\right)} \end{array}\right) \end{array}$$

and,

(7) $$\begin{array}{llll} \theta_{1} & \sim G_{0}\left(\theta_{1}\right)\; & & \;\\ \theta_{i}|\theta_{1}...\theta_{i-1} & \sim \frac{\tau }{\tau +i-1}G_{0}\left(\theta_{i}\right)+\overset{K}{\underset{l=1}{\sum }}n_{l}\frac{1}{\tau +i-1}\delta_{\Phi_{l}}\left(\theta_{i}\right),\;\text{for}\;i\geq 1.\; & & \;\\ & \; & \end{array}$$

Then the conditional posterior distribution for sampling *θ_i _*has the form

(8) $$\begin{array}{llll} p\left(\theta_{i}|\theta_{-i},s_{-i},X\right) & \propto q_{i,0}G_{i}\left(\theta_{i}\right)+\overset{n-1}{\underset{l=1,l\neq i}{\sum }}q_{i,l}\delta_{\theta_{l}}\left(\theta_{i}\right)\; & & \;\\ & =q_{i,0}G_{i}\left(\theta_{i}\right)+\overset{K}{\underset{l=1}{\sum }}n_{-i,l}q_{i,l}\delta_{\Phi_{l}}\left(\theta_{i}\right).\; & & \;\\ & \; & \end{array}$$

Thus the conditional posterior distribution for sampling Φ*_i _*has the form

(9) $$\begin{array}{llll} p(\Phi_{i}| & \Phi_{-i},s,X,\pi )\; & & \;\\ & \propto p(X_{m:s_{m}=s_{i}}|\Phi ,s,\pi \text{)}p\left(\Phi_{i}|\Phi_{-i},s,\pi \right)\; & & \;\\ & =G_{0}\underset{m:s_{m}=s_{i}}{\prod }\overset{L}{\underset{l=1}{\prod }}\frac{x_{ml}^{\alpha_{kl}-1}{\left(1-x_{ml}\right)}^{\beta_{kl}-1}}{B\left(\alpha_{kl},\beta_{kl}\right)}\; & & \;\\ & \; & \end{array}$$

It is obvious that *G*_0 _is not conjugate with *f*, so the integral *q*_*i*,0 _cannot be evaluated analytically and drawing samples from *G_i _*is also extremely challenging [21]. To overcome the difficulty, we adopt the "no-gaps" algorithm proposed in [22] to enable sampling from (8).

As to *τ*, it is useful to choose a weakly informative prior in many applications. If *τ *is assigned a gamma prior, its posterior becomes a simple function of *K*, then samples are easily drawn via an auxiliary variable method. For the convenience of sampling, we adopt the *τ ~ Gamma*(*a*, *b*) as the prior [9,20].

The final Gibbs sampling steps can be summarized by the following steps:

### Gibbs sampling for DPBMM

Iterate the following steps and for the *t*-th iteration:

1. For each sample *i*, re-sample *s_i _*according to (6) if $n_{s_{i}}>1$. In this case *k_-i _*= *K*. If $n_{s_{i}}=1$, then with probability 1 - 1/*K *leave *s_i _*unchanged. With probability 1/*K *rearrange *s *such that *s_i _*= *K*, then re-sample *s_i _*according to (6). But in this case *k_-i _*= *K *- 1.

2. For *i *= 1, ..., *K*, the posterior distribution for Φ*_i _*has the form as (9).

For *i *= *K *+ 1, ..., *n*, both prior and posterior distribution for Φ*_i _*are *G*_0_.

3. Sample *π *following (5) with $n_{k}=\sum_{i=1}^{n}\delta \left(s_{i},k\right)$.

4. Based on Step 1, we can get the value of *K*, then sample *τ*|*K*, *n *where *τ *~ *Gamma*(*a*, *b*).

Due to the large number of parameters, the initial values for parameters $\sigma_{\alpha }^{2}$ and $\sigma_{\beta }^{2}$ should be chosen carefully.

## Results

### Test on simulated data

We conducted simulations to test our proposed method. For the first case, the simulated data set is generated based on the model described in (2) with *K *= 4. The simulated dataset consists of 100 samples, each having 200 continuous response lying in the unit interval. The occurring probability of each cluster is set to {0.2, 0.3, 0.2, 0.3}. For each cluster, parameters *L_α_*, *L_β _*related to beta distribution in the model are generated randomly from Gaussian distributions with zero means and different variances. In order to systematically evaluate the clustering performance, the F metric that combines BCubed overall precision and recall [23] was implemented as suggested in [24]. Let {*c*} represent the real cluster label of samples and {*s*} represent the cluster assignment by clustering method, the correctness of the relation between sample *i *and *i' *is defined as *Ct*(*i*, *i'*) based on {*c*} and {*s*}.

(10) $$Ct\left(i,i^{\prime }\right)=\left\{\begin{array}{cc} 1 & \;\text{iff}\;c_{i}=c_{i^{\prime }}↔s_{i}=s_{i^{\prime }};\\ 0 & \;\text{otherwise}. \end{array}\right)$$

The overall precision *P *and recall *R *are defined as

(11) $$\begin{array}{c} R=Avg_{i}\left[Av{g_{i^{\prime }}}_{.c_{i}=c_{i^{\prime }}}\left[Ct\left(i,i^{\prime }\right)\right]\right]\\ P=Avg_{i}\left[Av{g_{i^{\prime }}}_{.s_{i}=s_{i^{\prime }}}\left[Ct\left(i,i^{\prime }\right)\right]\right] \end{array}$$

F metrics is used to evaluate the clustering result by combining *P *and *R *metrics.

(12) $$F\left(R,P\right)=\frac{1}{0.5/P+\left(1-0.5\right)/R}$$

**Figure 3:**
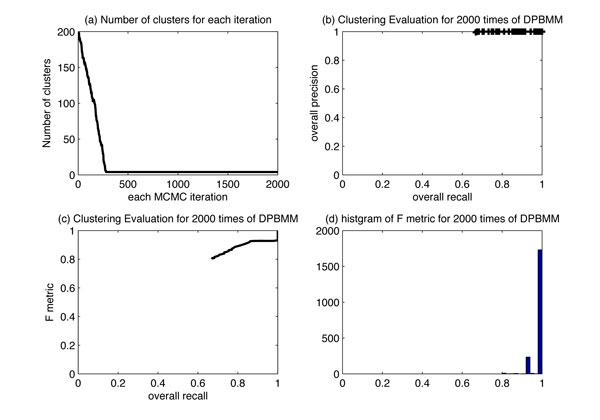
**Clustering evaluation on simulation data set**. The result is based on the simulated data with 4 dimensions. Figure 3(a) shows the number of clusters *k *in 2000 MCMC iterations. Figure 3(b) shows the overall precision vs. overall recall for 2000 times of DPBMM. The overall precision almost always stays at 1. Figure 3(c) shows the F metric vs. recall curve. Figure 3(d) shows the histogram of F metric results for 2000 times of DPBMM clustering.

Figure 3(a) illustrates the sampled number of clusters in each Gibbs sampling iteration for one time of DPBMM clustering. After 300 iterations of "burn-in" stage, the number of clusters stay at four. The uncovered cluster proportion is {0.19, 0.31, 0.19, 0.31}. Figure 3(b-d) show that for 2000 times of DPBMM clustering, F metric can come to one for most times.

**Figure 4:**
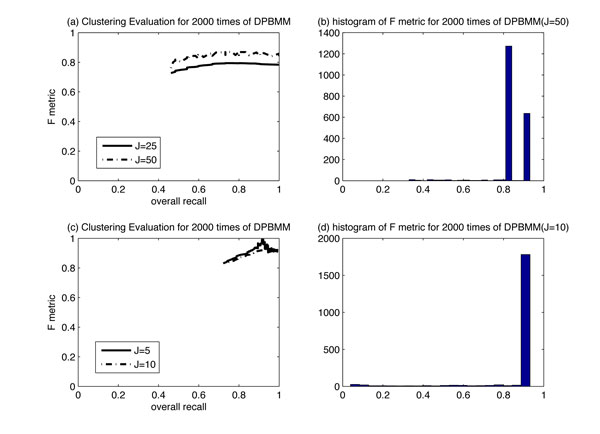
**Clustering evaluation based on different *J***. Figure 4(a) shows the F metric vs. recall curve of *J *∈ {25, 50} loci for case I. Figure 4(b) shows the histogram of F metric results with *J *= 50. Figure 4(c) shows the F metric vs. recall curve of different *J *∈ {5, 10} loci for Case II. Figure 4(d) shows the histogram of F metric results with *J *= 10.

For our second case, we used two simulated data set from [8]. The data set of Case I consists of 100 subjects, which mimics the real methylation data. Each subject has 1413 continuous responses lying in the unit interval. Each subject was a member of five classes, each cluster occurring with 0.2 probability. The clusters were defined by beta-distribution parameters for each of 1413 methylation loci that were autosomal and passed quality-assurance, obtained by fitting a beta model on each locus to one of the five data sets from our normal data: adult blood, newborn blood, placenta, lung/pleura, and everything else. The data set of Case II considered 100 subjects from four clusters. We compare the performance with RPMM method proposed in [8], with the same dimension reduction method employed. We order all the loci with respect to variance, and the *J *most variable loci are considered in the clustering algorithm. Table 1 and Table 2 summarizes the number of classes found with RPMM and with our proposed DPBMM for both Case I and Case II. For the cases considered, DPBMM obtained the correct *K *with *a priori ∞ *directly while the RPMM fitted finite mixture models for a range of possible values and chose the correct *K *by BIC statistic. The F metric vs. recall curve of *J *∈ {25, 50} loci for case I is shown in Figure 4(a). The histogram of F metric results with *J *= 50 is shown in Figure 4(b). The F metric vs. recall curve of different *J *∈ {5, 10} loci for Case II is shown in Figure 4(c). The histogram of F metric results with *J *= 10 is shown in Figure 4(d). For the above two cases, the more the number of loci are considered in the clustering, the better clustering performance we can get.

**Table 1:** Number of classes obtained for RPMM and DPBMM applied to simulated data (Case I: 5 classes).

Method	J	Median	Mean	SD
RPMM	25	8	7.7	2.0
	50	5	5.6	1.32

DPBMM	25	5	5.16	0.93
	50	5	5.29	1.43

**Table 2:** Number of classes obtained for RPMM and DPBMM applied to simulated data (Case II: 4 classes).

Method	J	Median	Mean	SD
RPMM	5	2	2.0	0.10
	10	2	2.4	2.38

DPBMM	5	7	6.9	1.04
	10	4	4.09	1.60

### Test on real data

We then applied our proposed DPBMM clustering on the GBM methylation microarray dataset in The Cancer Genome Atlas (TCGA). This dataset consists of 74 patients assayed on Illumina HumanMethylation450 array. Samples for DPBMM clustering analysis were selected to have clinical annotations. At last, 55 patients were left for consideration. Twenty-seven patients were alive at the time of last follow up, whereas twenty-eight patients experienced disease progression since last follow-up. The median follow up time was 198 days (range, 2-953 days). Each sample includes up to 485,577 CpG dinucleotides spanning gene-associated elements as well as intergenic regions. The associated detection P-value reported together with the methylation expression data is used as a quality control measure of probe performance. Following the probe excluding method in [25], the probes with detection P-values *>*0.01 in *>*10% of the samples are excluded from further consideration.

**Figure 5:**
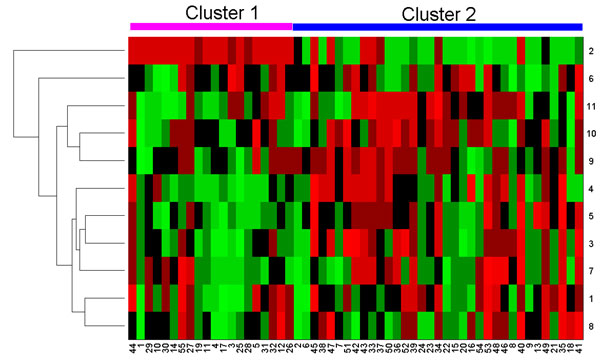
**Estimated clustering structure based on DPBMM and Hierarchical clustering**. 55 samples from TCGA are separated into two clusters on the basis of Illumina methylation expression array. The samples (columns) are arranged according to the estimated clusters by DPBMM while the locus (rows) according to hierarchical clustering.

**Figure 6:**
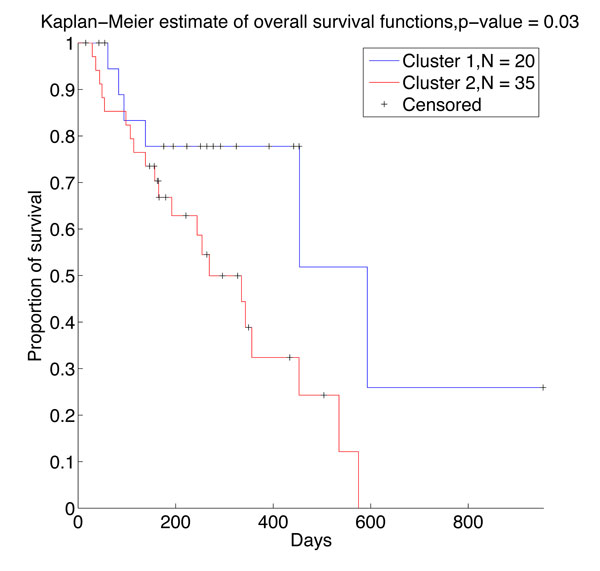
**Kaplan-Meier estimate of survival analysis based on uncovered structure of DPBMM method (J = 11)**. The figure shows the survival functions of the two clusters obtained based on the top 11 variable locus (P-value = 0.03) by DPBMM, which is more significant than the corresponding result of hierarchical clustering (P-value = 0.51).

Since the small sample, large dimensional property of methylation array, many loci in the data set have low variance and may not contribute to clustering. it is safer only to consider loci that change significantly [26]. Thus, those loci with low variance across all 55 samples were removed from the data sets which is also used by [8]. This also made the DPBMM clustering process computationally more tractable. In this paper, we only consider *J *∈ {1, 2, ..., 20} most variable loci for DPBMM clustering method since the number of samples is only 55. The selected top 20 variable loci are listed in Table S1 (see Additional file 1). DPBMM yields two clusters from the data for most *J*. Kaplan-Meier survival analysis are carried out based on the clustering results, and the P-values of Kaplan-Meier confidence for *J *∈ {1, 2, ..., 20} are shown in Table S2 (see Additional file 2). Among these, *J *= 11 gives the best P-value of 0.03. And the heatmap plot of *J *= 11 is shown in Figure 5, the Kaplan-Meier overall survival curve is shown in Figure 6. When *J *= 11, the clusters in GBM methylation array uncovered by DPBMM are statistically significant (P-value < 0.1). We also analyzed the survival of the two clusters uncovered by hierarchical clustering, but the clusters yielded are not statistically significant (P-value > 0.1).

**Figure 7:**
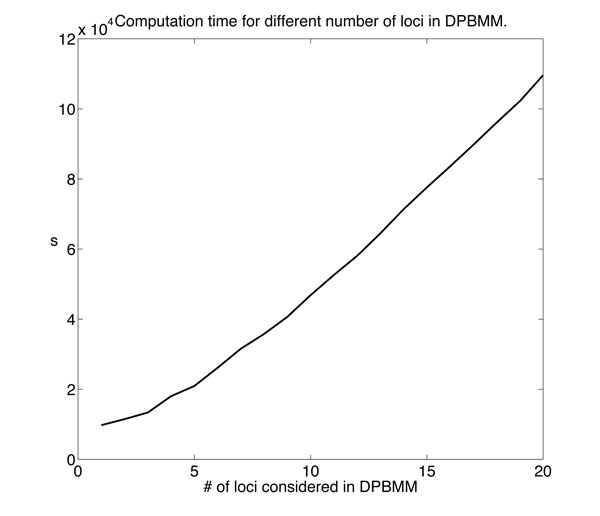
**The computation time resulting from the real data study for *J *∈ {1, 2, ..., 20}**. The figure shows the computation time resulting from the real data study for *J *∈ {1, 2, ..., 20}. It is carried out on a Linux based high-performance computer cluster. Each processing core is equipped with 2GB RAM. With the number of loci considered for DPBMM clustering, the computation time increases.

The computation time is always an issue for Gibbs sampling methods. Our simulation is carried out on a Linux based high-performance computer cluster. Each processing core is equipped with 2GB RAM. Figure 7 displays the computation time resulting from the real data study described before. The more loci considered for clustering, the more time the algorithm takes.

## Discussion

We discuss next a few distinct features of DPBMM. First, in accordance with the fact that "beta" values in DNA methylation array data fall in the range of zero to one, we assume mixtures of beta distribution for the data. It can provide more flexible shapes, thus can describe data of various types. This is different from traditional Gaussian mixture model based clustering methods such as K-means. Second, since most existing methods can not determine the number of clusters automatically, we adopted a Dirichlet process prior for cluster assignment. Thus, we get a non-conjugate Dirichlet process beta mixture model, whose parameters are hard to estimate. A Gibbs sampling and "no-gap" sampling solution is developed to overcome this difficulty. This is different from traditional parametric methods, whose result also relies on a model parameter, which is usually determined in a model selection process.

The limitation of the proposed methods are mainly as follows. First, the algorithm is based on Gibbs sampling, which is somewhat a resource-heavy MCMC method, therefore, the computation time is still heavy. Second, the model is computationally too slow to apply to methylation data of genome scale. We need to reduce the dimensionality to keep DPBMM computationally affordable.

In the future, it would be interesting to develop more effective dimension reduction method for DPBMM. It would also be interesting to integrate the information from different data sources such as gene expression and copy numbers variation into one model for cluster analysis.

## Conclusions

An infinite Dirichlet process beta mixture model was proposed to unveil the latent cluster structure from Illumina Infinium methylation profiles. By utilizing a Dirichlet process prior for cluster assignment, the number of clusters is determined. A Gibbs sampling and "no-gaps" sampling solution was developed to infer the relevant parameters automatically. The effectiveness and validity of the model and the proposed Gibbs sampler were evaluated on simulated data and on real data. The results demonstrated that DPBMM could yield the cluster structure automatically with better accuracy.

## Availability

MATLAB code is available at https://sites.google.com/site/bdpmmmethy/home.

## Competing interests

The authors declare that they have no competing interests.

## Authors' contributions

LZ, JM, and YH conceived the idea. LZ, JM, and YH worked out the detailed algorithms and derivations. LZ, JM and HL implemented the algorithm and performed the testing. LZ, JM, HL, and YH wrote the paper.

## Supplementary Material

Additional file 1 **Top 20 variable loci (ranked by variance through samples) selected from the methylation profiles of the 55 GBM samples**. Click here for file

Additional file 2 **The number of uncovered clusters and P-value of overall survival analysis for *J *∈ {1, 2, ..., 20}**. P-value is used to test the Kaplan-Meier confidence. Click here for file
