# Supplementary material for: A nonparametric Bayesian approach for clustering bisulfate-based DNA methylation profiles
Source: BMC Genomics. 2012 Oct 26;13(Suppl 6):S20. doi: 10.1186/1471-2164-13-S6-S20 (PMC3481479; doi:10.1186/1471-2164-13-S6-S20)
Supplement: Additional file 1 — Top 20 variable loci (ranked by variance through samples) selected from the methylation profiles of the 55 GBM samples. [file 1471-2164-13-S6-S20-S1.doc]

Table S1. Top 20 variable loci (by variance through samples) selected from the methylation profiles of the 55 GBM samples

| ILMNID | CHR | GENESYMBOL | COORDINATE | ILMNID | CHR | GENESYMBOL | COORDINATE |
| --- | --- | --- | --- | --- | --- | --- | --- |
| rs5931272 |  |  |  | rs2521373 |  |  |  |
| rs1416770 |  |  |  | rs5936512 |  |  |  |
| rs798149 |  |  |  | cg03686593 | 18 | FAM38B | 11149470 |
| rs5987737 |  |  |  | rs3818562 |  |  |  |
| rs6626309 |  |  |  | cg11738485 | 19 | HOOK2 | 12877000 |
| cg13686615 | 2 |  | 71503742 | rs4331560 |  |  |  |
| rs7746156 |  |  |  | cg14112997 | 22 | IL17RA | 17591088 |
| rs877309 |  |  |  | cg22336867 | 19 | LGALS7 | 39265241 |
| rs10796216 |  |  |  | cg11418607 | 15 |  | 67323243 |
| cg21272897 | 1 | HSPG2 | 22191453 | cg01759889 | X | FAM155B | 68725086 |
