# Supplementary material for: A nonparametric Bayesian approach for clustering bisulfate-based DNA methylation profiles
Source: BMC Genomics. 2012 Oct 26;13(Suppl 6):S20. doi: 10.1186/1471-2164-13-S6-S20 (PMC3481479; doi:10.1186/1471-2164-13-S6-S20)
Supplement: Additional file 2 — The number of uncovered clusters and P-value of overall survival analysis for J ∈ {1, 2, ..., 20}. P-value is used to test the Kaplan-Meier confidence. [file 1471-2164-13-S6-S20-S2.doc]

Table S2. The number of uncovered clusters and p-value of overall survival analysis for $J\in\{1,2,\cdots,20\}$. P-value is used to test the Kaplan-Meier confidence.

| J | K | p-value | J | K | p-value |
| --- | --- | --- | --- | --- | --- |
| 1 | 2 | 0.48 | 11 | 2 | 0.03 |
| 2 | 2 | 0.40 | 12 | 2 | 0.10 |
| 3 | 2 | 0.30 | 13 | 2 | 0.18 |
| 4 | 2 | 0.35 | 14 | 3 | 0.30* |
| 5 | 2 | 0.08 | 15 | 2 | 0.23 |
| 6 | 3 | 0.17* | 16 | 2 | 0.20 |
| 7 | 2 | 0.08 | 17 | 2 | 0.35 |
| 8 | 2 | 0.17 | 18 | 2 | 0.23 |
| 9 | 2 | 0.09 | 19 | 3 | 0.38* |
| 10 | 2 | 0.15 | 20 | 2 | 0.30 |

* p-value for the 3 clusters is the averaged pair-wise p-value.
